# Genomics for Arbovirus Surveillance: Considerations for Routine Use in Public Health Laboratories

**DOI:** 10.3390/v16081242

**Published:** 2024-08-02

**Authors:** Leandro Patiño, Andrea Denisse Benítez, Andrés Carrazco-Montalvo, Mary Regato-Arrata

**Affiliations:** 1 Instituto Nacional de Investigación en Salud Pública “Leopoldo Izquieta Pérez”, Dirección Técnica de Investigación, Desarrollo e Innovación, Guayaquil 090150, Ecuador; 2 Facultad Ciencias de la Vida, Escuela Superior Politécnica del Litoral, ESPOL, Campus Gustavo Galindo Km. 30.5 Vía Perimetral, Guayaquil 090902, Ecuador; 3 Instituto de Microbiología, Universidad San Francisco de Quito USFQ, Quito 170901, Ecuador; 4 Instituto Nacional de Investigación en Salud Pública “Leopoldo Izquieta Pérez”, Centro de Referencia Nacional de Genómica, Secuenciación y Bioinformática, Quito 170403, Ecuador; acarrazco@inspi.gob.ec; 5 Instituto Nacional de Investigación en Salud Pública “Leopoldo Izquieta Pérez”, Centro de Referencia Nacional de Virus Exantemáticos, Gastroentericos y Transmitidos por Vectores, Guayaquil 090150, Ecuador; mregato@inspi.gob.ec

**Keywords:** arbovirus, vector control, bioinformatics, public health, genome assembly, metagenomic sequencing

## Abstract

The emergence and re-emergence of arthropod-borne viruses is a public health threat. For routine surveillance in public health laboratories, cost-effective and reproducible methods are essential. In this review, we address the technical considerations of high-throughput sequencing methods (HTS) for arbovirus surveillance in national health laboratories, focusing on pre-sequencing, sequencing, and post-sequencing approaches, underlining the importance of robust wet and dry laboratory workflows for reproducible analysis. We aim to provide insights for researchers and clinicians interested in arbovirus, diagnosis, and surveillance by discussing current advances in sequencing methods and bioinformatics pipelines applied to arboviruses.

## 1. Introduction

Sensitivity, accuracy, reproducibility, time efficiency, cost-effectiveness, and validation are crucial criteria for choosing techniques for the diagnosis and surveillance of pathogens in public health laboratories [1,2,3]. Arthropod-borne viruses, also known as “arboviruses”, encompass a varied group transmitted by blood-feeding arthropods such as ticks, mosquitoes, sandflies, and biting midges. Dengue virus (DENV), Zika virus (ZIKV) (family *Flaviviridae*, genus *Flavivirus*), and chikungunya virus (CHIKV) (family *Togaviridae*, genus *Alphavirus*) are among the main arboviruses subject to routine surveillance in tropical and subtropical countries, where arthropod vectors thrive [4,5]. These viruses are involved in recurring large and expanding epidemic outbreaks and are a threat to temperate countries given the establishment of competent *Aedes* spp. populations associated with urbanization, human mobility, and climate change. DENV is estimated to cause 100–400 million cases and between 40,000 and 70,000 fatalities each year, with half of the world’s population at risk of infection [6]. Arbovirus infections in humans may present with a range of symptoms, such as fever, headache, muscle and joint pain, rash, myalgia, and fatigue. In some cases, the disease manifestations can lead to complications like shock syndrome or severe neurological damage, such as encephalitis, meningitis, paralysis, or even death [7,8].

Arboviruses surveillance is key for the early detection of seasonal outbreaks, new viral introductions, or even local evolution aimed at a rapid response, and the implementation of control measures [6]. National health laboratories (NHLs) play a crucial role in confirming suspected cases, genotyping the viruses, and monitoring changes in viral strains. Routine methods used at these laboratories include Enzyme-Linked Immunosorbent Assay (ELISA), virus isolation, plaque reduction neutralization test (PRNT), and nucleic acid tests (NATs)—particularly RT-qPCR, as most arboviruses are RNA viruses. However, understanding the true burden and evolution of arboviruses is hampered by reduced surveillance capacity using RNA-based tests, the high proportion of asymptomatic infections (e.g., up to 74% for DENV, ZIKV, and YFV), short viral incubation periods in humans (e.g., 2 to 14 days), low viremia in arthropod vectors, low RT-qPCR specificity, and the requirement of BSL-3 for improved viral detection [8,9,10,11,12].

High-throughput sequencing (HTS), also referred to as deep sequencing and next-generation sequencing, has surged as a disruptive technology for epidemic preparedness and outbreak response. Following SARS-CoV-2 surveillance, many public health laboratories have their installed capacity for applying it to arbovirus outbreak monitoring [13,14]. HTS allows for the genome sequencing of individuals from the same or different species simultaneously, for various sample matrices including cultured organisms, or directly from clinical or arthropod vectors. For arbovirus surveillance, HTS is a powerful genotyping method to determine whether the target pathogen is present in the sample. However, to take full advantage of HTS outputs, technical approaches must be carefully addressed before, during, and after sequencing. These include augmenting viral RNA, simultaneously sequencing different arboviruses or other virus families responsible for febrile illness, increasing the depth of coverage, and looking for mutation patterns with evolutionary significance [15,16,17]

This review discusses published information on HTS methods employed for detecting arboviral infection in humans and vector samples, focusing on pre-sequencing, sequencing, and post-sequencing approaches for NHLs.

## 2. Arbovirus Pre-Sequencing Considerations

### 2.1. Sample Collection and Storage

Sample collection and the storage of specimens for arbovirus surveillance and research are crucial for monitoring and understanding the epidemiology of these viruses. It might involve collecting entire arthropods such as mosquitos and ticks, vertebrates’ blood, serum, and other tissues. Sample storage will depend on the study objectives and available resources. Common methods for storing arthropods include the use of dry ice, liquid nitrogen, ethanol, and RNA-stabilizing buffer aimed to preserve viral RNA [18] and inactivate dangerous pathogens at the same time. Blood samples are collected preferentially in EDTA or without additives for serum separation by centrifugation. The sample type most used in NHLs is serum samples, as it can be used either for serological or PCR-based analysis [19]. In contrast to serum samples, whole blood has been shown to remain positive for weeks to months and could be a valuable sample type for genomic analysis [20]. Keeping a cold chain for transporting samples from remote locations is a challenge for NHLs [21]. RNA-stabilizing buffers and FTA^®^ (Flinders Technology Associates) classic cards are a useful alternative for tackling this issue. FTA cards are cotton-based cellulose paper containing a particular blend of denaturants and protecting agents. This chemical mixture degrades cells and proteins and inhibits bacterial development while protecting nucleic acids from free radical damage. The matrix traps RNA, maintaining its stability, while undesirable elements are washed away. FTA^®^ cards have been widely used for numerous samples such as blood, tissues, and serum [22].

### 2.2. Virus Enrichment

Some arboviruses such as ZIKV are known to have a low viral load or, in some cases, the infecting arbovirus could be detected with a low Ct [23] (cycle threshold at which a target nucleic acid is identified by q-PCR). In these circumstances, virus enrichment could be required before sequencing. One of the most common methods for arbovirus enrichment involves growing and propagating these viruses in cell cultures. It is commonly approached using Vero (African green monkey kidney cells) or C6/36 (*Aedes albopictus* mosquito cells), and various other mammalian cell lines depending on the virus in question [24]. Arbovirus growth in cell culture could take up to two weeks and requires specific aseptic areas and dedicated personnel. In addition, growing some arboviruses (CHIKV, Japanese encephalitis virus—JEV, West Nile virus—WNV) requires BSL-3 facilities, which are not available for all NHLs, especially in low- and middle-income countries, cell culture could also have low specificity for certain arbovirus species [21,25,26].

Ultracentrifugation-based methods could also be used for viral enrichment. Several ultracentrifugation methods described elsewhere include conventional ultracentrifugation, aimed at pellet viruses, separating them from other components in the sample, and the addition of polyethylene glycol (PEG) in a previous centrifugation increases virus precipitation [27,28]. Differential centrifugation involves spinning the sample at different speeds to separate particles based on their size and density. Gradient centrifugation with cesium chloride or tangential flow filtration has been used to separate viruses from other components based on size [3,29,30,31].

Additional methods described for viral enrichment include membrane filtration, using specific pore sizes to physically separate viruses from larger particles in the sample and concentrating the pathogens in the filtered solution. Also, immunomagnetic separation consists of magnetic beads coated with antibodies specific to the virus of interest, where a magnetic device is used to isolate the virus bound to beads from the rest of the sample [32].

### 2.3. Nucleic Acid Isolation

RNA can be isolated from a viral culture or directly from clinical samples of interest. Commercial kits are available for isolating RNA, and sequencing requires those that isolate highly pure RNA. Common nucleic acid isolation methods for genomic analysis are based on silica columns or magnetic beads using automatized instruments. To ensure the integrity of the RNA, the inactivation of RNases is a primary concern, this can be achieved by treating water with diethyl pyrocarbonate (DEPC) or by employing commercial RNase inhibitors [33]. It is highly recommended to work within a dedicated RNAse-free environment, using RNAse-free filter tips and disposable plasticware to prevent any potential RNA contamination. Maintaining a cold rack for reagent mixtures is recommended to preserve their quality. RNA isolation is usually complemented with the use of DNase enzymes to degrade co-isolated host DNA (from cell culture, host, or vector samples) which could potentially compete for the assignment of reads during sequencing.

Besides viral growth or ultracentrifugation, nucleic acid isolated from clinical or host samples can be enriched by several methods as described below.

### 2.4. Nucleic Acid Enrichment

#### 2.4.1. Nucleic Acid Capture by Hybridization

Also named targeted sequence capture (TSC), it involves the use of probes or baits to isolate target viral sequences from background nucleic acids. TSC increases viral reads and [34,35] offers advantages including the following: specificity, as the probes are designed to be highly specific to the target regions; flexibility, as the probes can be customized for different viruses; high coverage, due to increased sensitivity for detecting the target genome; and cost-effectiveness. An example is VirCapSeq-VERT, a commercial method that targets vertebrate viral genomes of 34 virus families (both DNA and RNA viruses), utilizing approximately 2 million capture probes [34,35]. The TSC method has a few limitations. An important limitation is that TSC panels may not effectively enrich highly divergent or novel viruses that are not included in the panel or do not share similarities with the covered viruses, which could be the case of the less-explored animal virome. Additionally, differences in viral loads may lead to biases in the representation of viral reads, for instance, viruses with high loads can be overrepresented in pooled samples which results in skewed data [35].

#### 2.4.2. Nucleic Acid Amplification

Nucleic acid amplification may be required to generate sufficient material for sequencing. PCR can provide both target enrichment and amplification in a single step and is relatively cheap, available, and fast compared with other methods. Most of these methods aim to amplify the genome of the organism of interest.

One of the most common methods for nucleic acid enrichment is using a tiling amplicon scheme that involves the use of primer pairs that amplify small, contiguous, or overlapping amplicons of the viral genome of interest. Primers can be designed for specific viruses or virus families and optimized for different sequencing platforms. Tiling amplicons provide high-resolution information by systematically covering every base pair in the target region. This makes it well suited for studying fine-scale genomic features [17].

Spiked primer enrichment is another method that, in combination with random primers, is used for the genome amplification of different virus families at the same time. The term “spiked” refers to the addition of these primers to the sequencing library. This method uses primers designed to target specific genes of the virus of interest. These primers “spike” the target genome, and when it is subjected to PCR, they selectively amplify specific regions of the virus of interest [36].

Multiplex PCR that targets multiple viruses is another strategy. Using a combination of virus or strain-specific primers, multiple viral genomes can be generated in a single PCR reaction [17].

Rolling circle amplification (RCA) is an isothermal multiple displacement amplification method used to obtain several copies of DNA or RNA from a circular template. It is particularly useful for enriching viral genomes in a sample, as many viruses possess circular or single-stranded genomes. RCA involves the use of random hexamers or specific primers, and specific polymerases (such as phi29) that possess strand displacement and proof-reading activity and can generate long synthesis products, which allows for the continuous copying of the template without the need for thermal cycling. RCA offers several advantages, including its sensitivity to detect low-concentration viruses, rapid completion within a few hours, and cost-effectiveness. However, there are some drawbacks to consider, such as the need for careful optimization of the reaction, it lacks high sensitivity and the potential for non-specific amplification. Despite these limitations, RCA remains a valuable method for virus enrichment, providing a sensitive and efficient means to detect viruses in samples with low viral concentrations [2].

Sequence-Independent Single-Primer Amplification (SISPA) is a nucleic acid amplification technique that differs from the others as it does not require previous knowledge of the pathogen sequence. It employs random primers on the 3’ end and a defined tag on the 5’ end, which will be used for subsequent amplification. The advantages of this method include the detection of known and unknown viruses in a wide range of samples and the ability to detect mixed viral infections and new viral species. Given that viral communities lack conserved molecular markers, unlike bacterial (16S) and fungal communities (internal transcriber spacer—ITS), random priming is a good strategy in viral metagenomics. A problem that may be encountered with this method is random priming to bacterial and host reads, which can affect the diversity of sequences found in the library [2,30,34,37].

## 3. Arbovirus Sequencing Considerations

### 3.1. Sequencing Methods for Arbovirus Surveillance

The choice of a sequencing method for arbovirus surveillance will depend on laboratory equipment, resources, sequencing instruments, and bioinformatics background. The first method for sequencing the arbovirus whole genome was Sanger sequencing [38], a first-generation sequencing method that involves the use of radioactive or fluorescently labeled dideoxy nucleotides, which lead to chain termination. Sanger sequencing only sequences a single and short DNA fragment at a time; however, it has 99.99% accuracy [39]. A bioinformatics background is not mandatory for processing data resulting from this process, as the methods available for assembling genomes are customized.

HTS for arbovirus sequencing has been reported since 2010, e.g., Bishop-Lilly (2010) and Cruz (2016) [40,41]. A comprehensive revision of the HTS platforms and sequencing technologies has been presented by Slatko et al. and Hu et al. [39,42]. Current HTS includes second- and third-generation Sequencing (SGS or TGS) platforms. SGS platforms currently used for the genome sequencing of arbovirus include the following: SOLiD, based on sequencing by ligation; Illumina, which uses sequencing by synthesis; and Ion Torrent, a semiconductor-based platform. The BGISEQ platforms based on DNA nanoball (DNB) and probe anchor synthesis technology have not been reported yet for arbovirus sequencing. Of the SGS methods tested for arbovirus HTS, Illumina offers a higher level of accuracy, enabling the precise sequencing of genomes from new pathogens. Illumina and Ion Torrent are considered more practical methods due to their reduced cost per base and larger outputs when compared to 454 and SOLID systems, both of which are no longer widely used in the field [29,43,44,45,46]. TGS platforms used for arbovirus sequencing include PacBio and Oxford Nanopore, both based on semiconductor technology. An advantage of TGS is the real-time detection of nucleotide incorporation events during the replication of single DNA molecules by DNA polymerase [44,47]. Oxford Nanopore Technology (ONT) has portable instruments the size of a stapler or a mobile phone, which have been used in the field using a mobile laboratory for Zika surveillance [47,48,49,50].

Illumina and ONT are the most common platforms used up to date for arbovirus sequencing. They use sequence-dependent (targeted) and sequence-independent (untargeted) approaches, proving that high-quality full viral genome sequences may be produced in 1–2 days during viral outbreak events [17,51,52,53,54,55].

**Figure 1:**
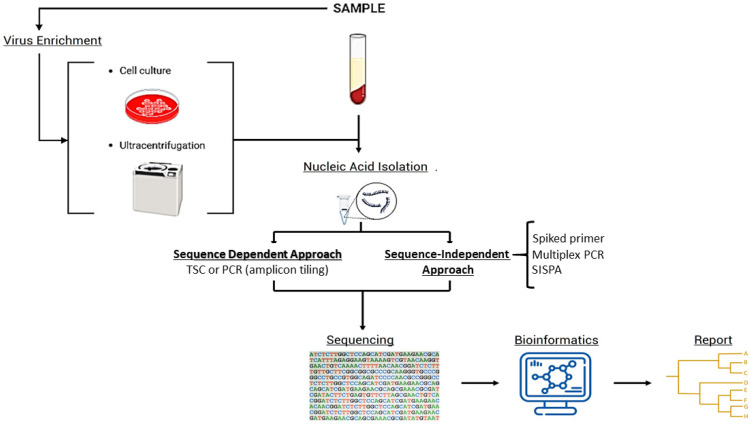
Schematic diagram of different approaches that could be used for arbovirus HTS workflow for optimal assemblies.

Arbovirus genomes can be sequenced following sequence dependent and sequences ondependent approaches. A schematic diagram of different approaches for viral sequencing that could be useful for arboviruses is shown in Figure 1. Table 1 highlights examples of previous research where different sequencing methods were employed, and where the bioinformatics procedures were applied.

#### 3.1.1. Sequence-Dependent Approach

These approaches allow for the targeted amplification of virus species, genera, or families. They include methods based on PCR and methods based on TSC. In relation to PCR-based methods, amplicon tiling is the most common approach. It uses reverse transcription followed by conventional PCR to generate amplicons that overlap by approximately 100 bp. Quick et al. (2017) employed an amplicon tiling strategy to produce comprehensive sequence coverage of ZIKV genomes. The authors developed a web-based primer design tool called primalscheme (latest version v1.4.1), which provides a complete pipeline for the development of efficient multiplex primer schemes for any virus. The software helps to design the primers, the length of the amplicon, and the overlapping section of each amplicon. For the 11 kb viral genome of Zika, scientists designed 35 primer pairs, each pair amplifying products of about 400 nT in length with a 100 nT overlap [17]. Vogels et al. recently proposed a pan-serotype HTS method for DENV [59].

Illumina has developed the commercial kit “Viral Surveillance Panel”, aimed to detect 66 viruses classified as “Important risks to public health” by the World Health Organization including arboviruses such as chikungunya, Zika, and the four dengue serotypes.

#### 3.1.2. Sequence-Independent Approach

Also named agnostic methods [2], these approaches allow for metagenomic sequencing (the direct sequencing of all genetic material present in a sample), allowing for unbiased viral detection. Sequence-independent approaches can be conducted through the spiked primer enrichment method (MSSPE), which involves the use of primers that anneal randomly to the viral RNA during the step of reverse transcription (cDNA synthesis) of the library preparation. MSSPE can be used with different sequencing platforms and has been shown to be efficient for arbovirus sequencing [36]. Several metagenomic methods for arboviruses have been described. For example, Jerome et al. (2019) demonstrated the potential of this method for the diagnosis of viral infections in febrile returning travelers using plasma samples. A total of six human viruses were detected including DENV and CHIKV. The study showed that metagenomics is an effective diagnostic tool, with a higher sensitivity and a faster turnaround time compared to conventional tests [43]. Another example was presented by Batovska et al. (2017), who employed a non-targeted detection approach with unamplified RNA/cDNA hybrids for library preparation and successfully detected and characterized the Ross River virus (RRV) from an infected mosquito [16].

Batovska et al. (2022) also focused on metatranscriptomic sequencing for arbovirus surveillance from field-collected mosquitoes, employing Illumina technology. The authors demonstrated the untargeted nature of metatranscriptomic sequencing, as it enables the detection of both known and novel viruses in mosquitoes, as well as other organisms of interest such as parasites, bacteria, and fungi [60].

Metatranscriptomics—the sequencing of all RNA isolated from a sample—was also used in research developed by [4]. The authors successfully reconstructed the complete genome of the ZIKV, including the 5 and 3 UTR regions, which would not have been possible using standard approaches. 

## 4. Arbovirus, Post-Sequencing Consideration

### Bioinformatic Analysis and Workflows for Arbovirus Sequencing Outputs

High-throughput data handling includes quality control, trimming, assembly, annotating, and displaying biological data of large volumes of sequencing data. It requires significant computational analysis and bioinformatics knowledge [15]. Several bioinformatics tools, algorithms, and pipelines have been developed depending on the sequencing platform and the type of analysis required, whether the data belong to a single organism or a mixed sample. A general workflow includes the steps described as follows:

*(I)* Quality control, the initial step, which includes evaluating statistical metrics and scores of sequences to assure accurate downstream analysis. *(II)* Precision trimming, cleaning, and improving the sequencing data by eliminating adapter sequences, discarding low-quality reads, filtering out contaminants, and removing low-quality segments and unnecessary elements. This optimizes the data for further analysis and ensures reliable and accurate results [57]. *(III)* Assembly, the process of reconstructing the complete genome sequence from fragments of shorter sequences, and there are two ways: **A**. Mapping assembly: sequences alignment with a previously known reference genome. This allows for the identification of variations between the sequenced individual and the reference, including mutations, insertions, or deletions. The resulting alignment data are stored in Binary Alignment Map (BAM) files, which are the compact and indexable binary representation of Sequence Alignment Map (SAM) files. This approach may not be suitable for highly variable genomic regions or genomes that differ significantly from the reference genome [59]. **B**. De novo assembly: involves genome assembly without the need for prior reference. Overlapping sequences assemble into longer fragments (contigs). This method is particularly useful when a reference genome is not available or when working with non-model species or very large genomes [42]. *(IV)* Taxonomic profiling: assigning taxonomic identities to sequences, allowing the species present in a the sample to be identified and classified. Additional analysis may include functional annotation to explore information about gene function and biological processes, aimed at a better understanding of the molecular mechanisms behind biological events, directing future experimental studies, and statistical analysis [23,41,42,57,58].

There are several ways to analyze sequencing data in genomic and metagenomic research, providing researchers with varying degrees of control, flexibility, and personalization. The use of locally installed software packages is a typical solution, as researchers can have direct control over the analysis process and adjust it to their individual needs by installing and configuring bioinformatics tools on their own workstations. This requires technical expertise, computational resources, and time for installation, maintenance, and updates (Table A1). Custom scripting is another method in which researchers create their own scripts or workflows using programming languages (such as Python, R, or Perl). This method offers the most flexibility and customization, allowing users to employ specialized analytical processes, though it requires advanced programming skills and a significant amount of time and effort to develop and maintain the scripts [15].

Software bundles that combine tools and processes into one package, like the Bioconductor suite for R, and EPI2ME, offer solutions for enhanced analysis. Researchers use bioinformatics tools and pipelines to efficiently organize the processing, analysis, and interpretation of large amounts of sequencing data. Another alternative is web-based tools that provide end-to-end bioinformatics pipelines. These platforms combine a variety of computational tools, methods, and databases, which allows researchers to perform a variety of analyses, including data quality control, read alignment, variant calling, functional annotation, and visualization (Table A2). However, a disadvantage is that some pre-designed tools often lack updates, which could affect genetic variant assignment, due to the use of outdated databases; ultimately, and they may transition to paid software models, limiting accessibility. Specific pipelines have also been developed for DENGV, and for an example of this, see Mendes et al., 2020 [61].

## 5. Discussion

Incorporating genomics into public health initiatives for arboviral research and surveillance has enormous potential to reduce their impact. The World Health Organization has acknowledged the value of genomics methods in understanding transmission dynamics, developing global genomic surveillance strategies, and responding to emerging and re-emerging outbreaks. The COVID-19 pandemic was an important turning point in genomic monitoring, exhibiting the exceptional speed of pathogen characterization and data sharing. National health laboratories implemented or strengthened their genomic capacity which could be harnessed to increase the genomic surveillance of pathogens of concern [9,57].

HTS has several advantages for diagnosing clinical cases as reviewed by Lui et al., 2023 [62]. Genome sequencing is a valuable complement to antigen or NAT. Genome analysis allows for viral characterization to the level of genotypes which is crucial to investigate the implication of viral variants causing outbreaks or severe illness. For example, DENV is known to have four serotypes, and each of them is genetically subdivided into genotypes; while RT-qPCR detects serotypes, sequencing leads to genotypes. Serotyping at least 10% of positive DENV cases nationwide is a common algorithm of NHLs, and in comparison with q-PCR data, HTS allows for the detection of genetic variation through the genome. In circumstances where the nucleic acid test fails to detect the pathogens targeted—usually three multiplexed viruses in RT-qPCR—agnostic metagenomic methods would allow for the identification of the suspected pathogens or any other viruses causing the disease. High throughput and the depth of coverage obtained by HTS allow for the detection of minor variants down to 1–5%. These features provide new avenues to study viral populations at the individual (patient) level which would help to better understand the rise of new viral variants or the biological meaning of viral intra-host diversity [63]. 

For epidemiological studies, HTS has shown to be crucial in identifying early arboviral outbreaks, detecting specific viral strains, tracking their spread to new geographic areas, and identifying the local emergence of novel strains. For example, WGS has been used for studying the ZIKV in Brazil, where it has been detected since 2015 [64]. Genomic analyses identified the Asian lineage as the driver of the epidemic outbreaks in the Americas and allowed for the detection of the African lineages in enzootic cycles between non-human primates and mosquitoes [65]. It also allowed for the identification of the recent introduction to the Americas of the DENV-2 cosmopolitan genotype lineage [66] and a better understanding of the dynamics of circulating lineages/genotypes [67].

Arbovirus genome sequencing could also contribute to strengthening One Health initiatives and early warning systems for arbovirus infection. In relation to the One Health approach, arboviruses often have complex transmission cycles involving wildlife and zoonotic reservoirs [68]. Genome sequencing facilitates studying their diversity in different hosts including vectors. Ongoing initiatives aim to establish early warning systems for arbovirus infection in some countries of the Mediterranean (Serbia) and Black Sea region (Georgia) and Brazil. These efforts seek to integrate the surveillance of human, animal, entomological, and environmental sectors. Incorporating genomics into intersectoral surveillance would leverage these programs providing valuable genetic information [69,70], useful for understanding and predicting potential changes in arbovirus behavior and implementing timely interventions [69,70,71].

Similar to SARS-CoV-2, sharing arbovirus genome sequencing data on a global scale would create opportunities for collaboration among researchers and public health agencies. This collective effort could enhance our ability to respond effectively to arbovirus threats and identify specific viral proteins that could serve as targets for vaccine development or potential targets for antiviral drug development. An example of this is the EpiArbo initiative, launched by GISAID. It provides an online platform for researchers worldwide to share genomic data on arboviruses, helping to improve surveillance, diagnostics, treatments, and vaccine development. With open access to data and a global network of collaborators, EpiArbo strengthens the global scientific community to stay ahead of arbovirus outbreaks and limit their impact.

No single sequencing strategy is ideal for all situations, and a combination of strategies may be required to obtain the best outcomes. It is important to perform a careful standardization of the protocol to take full advantage of genome sequencing [36,72]. Further optimization and standardization of sample preparation methods are needed to ensure reproducibility and comparability across laboratories [73]. This will enable the development of robust protocols applicable in diverse settings and facilitate molecular epidemiology analysis.

The development of user-friendly bioinformatics tools and pipelines specifically designed for arboviral data analysis is necessary. These tools should address the challenges associated with data processing, quality control, variant calling, and data integration, hence facilitating accurate and reproducible analysis across different research groups. Including bioinformatics analysis that looks for significance in mutations in consensus sequences and at the viral population level will have a major contribution to understanding arbovirus evolution [74,75]. In the Americas, important efforts are being made to standardize wet and dry laboratory protocols through PAHO initiatives such as the The Arbovirus Diagnosis Laboratory Network of the Americas and the Arbovirus geomic Surveillance Network, RELDA and Vigenda by its Spanish acronym (The Arbovirus Diagnosis Laboratory Network of the Americas (RELDA): https://www.paho.org/en/topics/dengue/arbovirus-diagnosis-laboratory-network-americas-relda, accessed on 28/7/2024). 

From an NHL perspective, HTS does not replace conventional virological methods for arbovirus surveillance. However, it does represent a powerful modern method to complement and enhance these activities. Technical limitations of HTS include workflows of >1 day and the lack of interlaboratory standardization both for wet and dry laboratories. Sequence quality is dependent on viral load, and false-negative results could be obtained from unscreened samples. Laboratory budgets might also restrict HTS implementation as it requires substantial initial investment and high operational costs. Equipment and reagents are costly, with prices increasing according to the sequencing platform and geographical location. Manufacturers are located in high-income countries, and reagents and supply importation to low- and middle-income countries increases costs twice or three times in comparison with countries from the northern hemisphere; also, delivery times are longer. Data analysis also requires hiring personnel specialized in bioinformatics and escalating computing infrastructure for data storage and processing.

Taking limitations into account, improvements in sequencing technologies and the development of cost-effective platforms will facilitate broader access to comprehensive genomic data, particularly in regions with limited resources. This will aid in the surveillance and monitoring of arboviral outbreaks. Currently, all sequencer manufacturers are developing portable instruments, reducing equipment and reagent costs, which could contribute to NGS democratization. Investment in capacity building and training programs in low- and middle-income countries is essential to ensure that researchers, clinicians, and public health professionals have the necessary skills and knowledge to effectively apply molecular methods and interpret sequencing data for arboviral detection and surveillance. Established external quality control for wet and dry laboratories would increase confidence and the reproducibility of the results. Adopting HTS for routine surveillance of arboviruses would enhance the understanding of transmission dynamics, arboviral genetic evolution and improve diagnostic tests, allowing for rapid responses and containment measures to prevent widespread infections.

## Figures and Tables

**Table 1:** Sequencing strategies used for arboviruses for targeted sequencing and/or metagenomics, and the bioinformatic workflows or pipelines employed for analysis.

Arbovirus	Depletion or Enrichment Methods	Genomic Scope (Sequence- Dependent “sd” or Sequence- Independent “si”)	Sequencing Platform	Bioinformatic Workflow/Pipeline	Reference	Advantages	Disadvantages
DENV, ZIKV, CHIKV	Not used	Metagenomics complete viral genomes (si)	NextSeq 2000	Pre-processing (FastQC, TrimGalore, and Fastp) Taxonomic classification and removal of host not targeted reads (Kraken2) De novo assembly (SPAdes) Taxonomic classification based on protein identity (Diamond BLASTx) Phylogenetic analysis (MAFFT, IQ-TREE, and FigTree)	[8]	High specificity of viral identification and the unbiased nature of viral detection, full annotation of all viruses present in the tested sample was enabled.	Not sensitive enough to detect low levels of viral nucleic acids, may also detect non-infectious viral particles or viral nucleic acids from previous infections.
Simultaneous detection of vector-borne viruses	Metagenomic sequencing with spiked primer enrichment (MSSPE)	Metagenomic (sd)	MiSeq and MinION MK1B or GridION X5	Identification of viral reads Removal of spiked primers Duplicate reads removed (Prinseq) Mapping and assembly	[36]	Simple, low cost, high sensitivity, and broad detection capability, fast and deployable.	Prior knowledge of the target viruses is required.
DENV, CHIKV	Not used	Metagenomic next-generation sequencing (si)	MiSeq	Pre-processing (Trim_galore and Prinseq) Taxonomic classification (Diamond BLASTx) De novo assembly and mapping (SPAdes, dipSPAdes, and Tanoti) Visualization (Krona plots and BLAST tables)	[43]	Ability to detect new and emerging pathogens, potential for resistance screening and phyloepidemiological analyses. Rapid and accurate identification of viral infections in febrile returning travelers. This is essential for timely treatment and the prevention of further transmission.	Limited detection of DNA viruses, enrichment or depletion methods may be necessary.
ZIKV, CHIKV	Multiplex tiling PCR	Metagenomic amplicon sequencing (sd)	MinION	Demultiplexing (demultiplex.py script or Metrichor) Mapping to reference genome (BWAmem and SAMtools view) Primer trimming (align_trim.py script) Variant calling (nanopolish variants) Consensus generation (margin_cons.py script) Visualization of variant calls and frequencies (vcfextract.py and pdf_tree.py)	[17]	Inexpensive and convenient lab method, multiplex PCR reduces the cost of reagents and minimizes potential sources of laboratory error.	Method is not suitable for the discovery of new viruses or for sequencing highly diverse or recombinant viruses because primer schemes are virus-genome-specific.
			MiSeq	Primer trimming (Trimmomatic) Alignment (Novoalign) Statistics (SAMtools)			
YBV1, YPLV3, YSLV1 ^a^	Random amplification with octamer primers	Metagenomics complete viral genomes (si)	MiSeq	Pre-processing (Cutadapt and Prinseq-lite) De novo assemblies (Ray Meta) Contig/scaffold assembly (Cap3) Taxonomy classification (iterative BLAST search against the NCBI nt database) Verification of viral contig sequences (in-house reference-mapping pipeline NGS_Mapper) Visualization and curation (Geneious R10 and IGV) Annotations (Blastp, TMHMM, and EVEs) Phylogenetic analysis (MEGA)	[55]	The study identified novel viral genomes in mosquitoes in the Republic of Korea. The research lays the groundwork for developing a map of complete mosquito viromes that is closely cross-referenced to the population statistics of every mosquito species in the ROK. Eventually, this map might be enlarged to create a global database.	The study did not investigate the potential pathogenicity or transmission of the identified viral genomes.
CCHFV, RVFV, DUGV, NSDV, MIDV, WSLV ^b^	SISPA	Whole-genome sequencing (si)	MinION	Pre-processing (Mk1C Guppy) Demultiplexing and adaptor removal (Porechop) Mapping and genome assembly (KMA and Minimap2)	[56]	Allows for a broad enrichment of viral genomic RNAs without the need for virus-specific whole-genome PCRs. Useful for the detection and characterization of viruses in samples with unknown or complex viral populations.	Unclassified reads cannot be distinguished when multiple samples of the same virus but of different origins have been sequenced in the same run (e.g., CCHFV from a tick and CCHFV from cell culture supernatant).
DENV types 1–4, CHIKV	Not used	Whole-genome sequencing of targeted virus (sd)	MiSeq	Pre-processing (ngs_mapper pipeline and Trimmomatic) Mapping and remapping to reference (BWA–Maximal Exact Matches) Assembly processing (samtools and in-house basecaller.py) Statistics (matplotlib) Phylogenetic analysis (Phyml)	[54]	The study presents one of the most detailed descriptions of dengue and chikungunya illnesses in Ecuador’s southern coast.	Researchers only questioned the immediate neighbors of the original index case, which may not be typical of the entire community living within 200 m.
NPV ^c^	Cell culture and random hexamer primers	Whole-genome sequencing (si)	MiSeq and Sanger sequencing	Mapping with phleboviruses and comparison against amino acid sequence libraries (BLAST) Genome assembly and annotations (Geneious) Phylogenetic analysis (MEGA)	[57]	The broad reverse transcription PCR screening allowed us to discard orthobunyaviruses, alphaviruses, and flaviviruses. The screening for sand fly-borne phleboviruses by targeting the RdRp gene using degenerate primers and phylogenetic analysis allowed us to classify the new virus.	This approach requires extensive laboratory work and expertise.
Pool of six viruses: MAYV, VEEV, CHIKV, ZIKV, VSV, and OROV ^d^	Ribosomal RNA depletion	Direct RNA sequencing (sd)	MinION	Pre-processing (Albacore) Mapping (Minimap2, Samtools) Read coverage calculation (Genomecov in BEDtools) Reference-based assembly (Racon)	[58]	Enables the detection of a complex population of RNA viruses simultaneously, fast and easy detection within 3 h.	Requires high amounts of virus, poly(A)-tail reaction is not only specific to viral RNA, but host RNA can also be a target, requires prior knowledge of a target nucleotide, this method may not find novel RNA viruses or coinfections.

^a^ Yongsan bunyavirus 1 (YBV1), Yongsan picorna-like virus 3 (YPLV3), Yongsan sobemo-like virus 1 (YSLV1). ^b^ Crimean–Congo hemorrhagic fever virus (CCHFV), Rift Valley fever virus (RVFV), Dugbe virus (DUGV), Nairobi sheep disease virus (NSDV), Middleburg virus (MIDV), and Wesselsbron virus (WSLV). ^c^ Ntepes virus (NPV). ^d^ Mayaro virus (MAYV), Venezuelan equine encephalitis virus (VEEV), Vesicular stomatitis Indiana virus (VSV), and Oropouche virus (OROV)5. Arbovirus, post-sequencing consideration.

## Appendix A

**Table A1:** Tools employed in genomics and metagenomics analyses.

Category	Tool Name	Description	NGS/TGS	Reference
**Pre-processing and Quality Control**	FastQC	Quality control analysis tool for high throughput sequencing data	NGS	[76]
	Nanoplot	Quality control analysis tool for ONT data	TGS	[77]
	Nanopolish	Software package for signal-level analysis of ONT data	TGS	[78]
	Porechop	Adapter trimmer for ONT reads	TGS	[79]
	Fitlong	Tool for filtering long reads by quality	TGS	https://github.com/rrwick/Filtlong#license (accessed on 24 July 2024)
	Trimmomatic	Read-trimming tool for Illumina NGS	NGS	[80]
	Cutadapt	Remove adapter sequences	NGS	[81]
**De novo genome assembly**	Flye	De novo assembler for single molecule sequencing reads using repeat graphs	TGS	[82]
	Canu	A single molecule sequence assembler for large and small genomes	TGS	[83]
	Unicycler	Hybrid assembly pipeline for bacterial genomes	NGS/TGS	[84]
	Spades	Genome assembler for genomes of regular and single-cell projects	NGS/TGS	[85]
	Medaka	Tool to create consensus sequences and variant calls from nanopore sequencing data	TGS	https://github.com/nanoporetech/medaka (accessed on 24 July 2024)
	Wtdbg2	De novo assembler for long noisy sequences, based on fuzzy Bruijn graphs	TGS	[86]
**Short-read mapping**	BWA	Maps short reads against reference genome	NGS	[87]
	STAR	RNA-seq aligner that can detect and align both spliced and unspliced reads	NGS	[88]
**Long-read mapping**	Minimap2	Pairwise aligner for genomic and spliced nucleotide sequences	TGS	[89]
	GraphMap	A highly sensitive and accurate mapper for long, error-prone reads	TGS	[90]
**Taxonomy and metagenomics**	Kraken2	Taxonomic sequence classification system	TGS	[91]
	PlasFlow	Software for prediction of plasmid sequences in metagenomic assemblies	TGS	[92]
	MetaPhIan2	Tool to profile structure and composition of microbial communities (bacteria, archaea, eukaryotes, and viruses) from metagenomic shotgun sequencing data with species-level resolution	NGS	[93]
	DIAMOND	Tool for rapidly searching large DNA or protein sequence databases for short sequences	NGS	[94]
**Visualization**	Krona	Interactively explore metagenomes and more from a web browser	NGS/TGS	[95]
	Phinch	Data visualization framework aimed at promoting novel explorations of large biological datasets	NGS/TGS	[96]
**Variant calling**	Medaka	Create a consensus sequence from ONT data	TGS	https://github.com/nanoporetech/medaka (accessed on 24 July 2024)
**Statistical analysis**	Bracken	Relative abundance estimation tool for single-level abundance using Kraken read classification output	TGS	[97]

**Table A2:** Web-based tools for virus analysis.

Web-Based Tools	Brief Description	Link	Reference
**BV-BRC**	Bacterial and Viral Bioinformatics Resource Center, an information system designed to support research on bacterial and viral infectious diseases. It allows single or multiple assemblers to be invoked to compare results	https://www.bv-brc.org/app/Assembly2 (accessed on 24 July 2024)	[98]
**CASTOR**	Provides an open access, collaborative and reproducible machine learning classifier that enables novel and accurate large-scale virus studies.	http://castor.bioinfo.uqam.ca (unable to access, returns an error message)	[99]
**EDGE**	Workflows for metagenomic analysis that are pre-configured and may be accessible via a webpage or a local installation. Supports long read and viral genome analysis	https://edgebioinformatics.org/ (accessed on 24 July 2024)	[100]
**Galaxy**	Platform that provides access to a wide range of tools for analyzing biological data which includes DNA and RNA sequencing, proteomics and metabolomics data	https://galaxyproject.org/ (accessed on 24 July 2024)	[101]
**Genome** **detective**	A web-based platform that enables the identification of organisms responsible for an outbreak and evolutionary information of pathogens crucial in understanding the unique phenotypes such as drug resistance, virulence and disease outcome.	http://www.genomedetective.com/app/ (accessed on 24 July 2024)	[75]
**IMG**	Enables comparative analysis of metagenomes registered on Genomes OnLine Database. Supports analysis of viral genomes through IMG/VR	https://img.jgi.doe.gov/ (accessed on 24 July 2024)	[102]
**Kaiju**	Fast and sensitive taxonomic classification of high-throughput sequencing reads from metagenomic whole genome sequencing or metatranscriptomics experiments	https://bioinformatics-centre.github.io/kaiju/ (accessed on 24 July 2024)	[103]
**Metavir 2**	Web server dedicated to the analysis of viral metagenomes (viromes)	http://metavir-meb.univ-bpclermont.fr/ (unable to access, returns an error message)	[104]
**MGnify**	Platform for analysis of European Nucleotide Archive (ENA)-based sequence and metadata. Provides pipelines for the detection, annotationa and taxonomic classification of viral contigs in metagenomic and metatranscriptomic assemblies.	https://www.ebi.ac.uk/metagenomics/ (accessed on 24 July 2024)	[105]
**NanoGalaxy**	Nanopore long-read sequencing data analysis in Galaxy	https://galaxyproject.org/use/nanogalaxy/ (accessed on 24 July 2024)	[106]
**Nextstrain**	A real-time pathogen evolution tracking platform that provides evolutionary information in the form of interactive visualizations. It has been used to track various arboviral epidemics globally.	https://nextstrain.org/ (accessed on 24 July 2024)	[107]
**VirusDetect**	Uses deep sequencing of small RNAs to identify both known and novel viruses in plants and animals. It performs both reference-guided and de novo assemblies of small RNA sequences and compares them to a curated virus reference database.	http://virusdetect.feilab.net/cgi-bin/virusdetect/index.cgi (accessed on 24 July 2024)	[108]
